# Research on the integral forming process of thin walled and thick mouth seamless gas cylinders

**DOI:** 10.1038/s41598-023-44377-z

**Published:** 2023-10-09

**Authors:** Chen Wang, Haofei Yu, Wang Tian, Chunjiang Zhao, Lianyun Jiang, Qiaofeng Bai

**Affiliations:** School of Mechanical Engineering, Taiyuan University of Science and Technology, Taiyuan, 030024 China

**Keywords:** Mechanical engineering, Metals and alloys

## Abstract

There is a considerable difference in wall thickness between the mouth and the cavity of thin-walled and thick-mouthed seamless gas cylinders, and the existing manufacturing processes are unable to effectively meet product requirements. To overcome such issue, a step-by-step boring-necking-spinning solution for gas cylinders was proposed, in which sufficient wall thickness is reserved for the mouth area of the cylinder blank, followed by necking-spinning to realize the overall forming of thin-walled, thick-mouthed seamless gas cylinders. The stress–strain distribution and geometric dimensional changes of gas cylinders during the spinning process were investigated by means of finite element simulation, and the effects of different process parameters on the stress and wall thickness of the bottle mouth were analyzed. Further, multi-objective optimization of the response surface model was performed using the NSGA-II algorithm to derive a set of optimal process parameters. Finally, the correctness of the simulation and optimization results was verified experimentally, and the expected geometry and optimal strain state of the gas cylinder were obtained. The newly developed processing solution represents a groundbreaking advancement in the manufacturing of thin-walled and thick-mouthed gas cylinders.

## Introduction

As human civilization advances and science and technology continue to develop, the impact of energy consumption and global warming has attracted significant attention worldwide. Consequently, research into alternative energy sources, including the transportation and storage of clean energy sources such as hydrogen and natural gas, has become a priority.

Gas cylinders are widely used in the storage and transportation of clean energy. Azeem et al.^1^ highlighted that a seamless gas cylinder liner, composed of a metal liner with a high-strength carbon fiber reinforced composite wrapped around the outer layer, is a promising option for high-pressure storage containers. Such design is advantageous owing to the lighter weight, increased strength, gas-tight properties, and adequate safety features.

Due to the limited volume of gas cylinders, gases are usually stored under high pressure. Such conditions require cylinders to be light in weight and large in volume, while ensuring sufficient strength. Therefore, the structural design of gas cylinders necessitates a thin wall thickness for the cavity to minimize weight. However, the mouth of the cylinder must have a sufficient thickness to meet the strength requirements of the thread. The development of the manufacturing process of thin-walled and thick-mounted cylinders, which can largely improve the storage efficiency of hydrogen, has also attracted considerable attention.

At present, a large number of domestic and international scholars have conducted research on gas cylinder-forming technology. The traditional production process of gas cylinders involves a stamping process to form the upper and lower head parts, respectively, followed by welding. Music et al.^2^ pointed out that coreless necking and spinning technology is widely used in the necking and forming process of axisymmetric rotary parts. Wong et al.^3^ showed that spinning technology, as the main production method for gas cylinders, fundamentally solves the problems of discontinuity, low strength, brittle cracking and stress concentration of weld seams in the traditional welding production of gas cylinders, and at the same time, spinning allows the production of parts with high mechanical properties and smooth surface finish. Wang et al.^4^ explored welded gas cylinders and found that residual stresses due to weld seams had a significant impact on strength, fatigue life, and gas tightness, thereby posing a considerable safety hazard to the cylinders.

Marini et al.^5^ claimed that the flow-forming process can be extensively adopted for production of thin-walled, high-precision tubular products. Wong et al.^6^ used a two-step forming process consisting of "bending" and flow forming. The method involved the flow of material along a mandrel to shape thin-walled cup-shaped parts, with the process employing two distinct profiles and axial rolling. Jin et al.^7^ researched a monolithic manufacturing process for large-diameter seamless steel cylinders. The monolithic forming of cylinders could be achieved by means of cold spinning of the cylindrical parts obtained by deep drawing. Through such means, the cylinder wall thickness uniformity was greatly improved and the dimensional accuracy was higher. By investigating the relationship between the microstructure and mechanical properties of gas cylinders, Li et al.^8^ improved the mechanical properties of gas cylinders based on the manufacturing process proposed by Jin et al.^7^. Zoghi and Fallahi Arezoodar^9^ manufactured pressure vessels using a necking spinning process, which essentially involved forming parts by applying the required force and displacement to the rotating blank by means of one or more forming rolls. Wang et al.^10^ claimed that a combination of stamping and deep drawing using steel plates could produce cylinder blanks as well as cups. Cylinder blanks prepared through such process had the advantages of uniform thickness, high strength, and light oxidation.

Music et al.^11^ reported that metal spinning can improve the surface finish and mechanical strength of molded parts compared with stamping. M.L. According to A et al.^12^, the utilization of numerical modeling via finite element flow formulation can effectively facilitate the comprehension and prediction of different modes of deformation during the end forming of thin-walled tubes.

Kuang et al.^13^ used Ansys finite element software to establish a three-dimensional finite element model of offset circular tube header spinning. Using the model, during three-dimensional non-axisymmetric spinning, the effects of metal flow, stress and strain distribution, spinning pressure, and different process parameters on the spinning results were investigated.

In analyzing the evolution of the material shape and thickness as well as the stress and strain distribution generated during the spinning process, Iguchi et al.^14^ used the dynamic explicit code DYNA-3D to analyze the spinning manufacturing process of motor vehicle exhaust system components to further understand the failure mechanisms such as fracture and buckling during the spinning process. The results could provide useful information for failure prediction during the actual spinning process.

Xia et al.^15^ proposed a finite element model for non-axisymmetric neck spinning using the finite element software MSC to obtain the transient Mises stress distribution in the contact zone between the roll and the billet, as well as the equivalent plastic strain after spinning. Further, numerical and experimental studies on the thickness distribution of the spun workpiece were conducted. Such studies provided reasonable suggestions for addressing the occurrence of excessively thin or thick wall thickness in the workpiece. Yao and Makoto^16^ conducted an experimental study on the near-axis spinning of tube ends and investigated the effects on thickness strain, torsion angle, spinning force and surface finish of aluminum products with the parameters of spinning pitch and diameter reduction. Through experiments and 3D finite element simulations, Yt et al.^17^ investigated the effect of neck length on crack generation during the spinning of SUS409, and determined the spinning conditions under which cracking would not occur based on the calculated damage values. Huang et al.^18^ explored the thickness distribution and outer profile of the tube necking spinning process using the finite element model established by the shell cell. that the research revealed that the thickness of the spun tube had a small amount of thickening with the spinning process, but there was no explanation given for the deformation in the material thickness. Hamed et al.^19^ established a three-dimensional finite element model of circular tube spin forming to investigate the strain distribution in different thickness layers of a tube during the spin-forming process. An increasing trend of strain in the middle layer thickness toward the free end was reported, which indicated an increase in the tube wall thickness. Biplov Kumar Roy et al.^20^ investigated the change rule of geometry and thickness during necking and spinning forming of tubes by combining experimental and numerical simulation.

Response surface methodology (RSM), also known as regression design, is a statistical test for optimizing stochastic processes. The purpose is to analyze the significance of each factor on the response value, to obtain a quantitative and reasonable regression equation, and to guide the engineering application. The central composite design is a commonly used response surface analysis method, which is proposed on the basis of two-level full-factor partial experimental design. Zhou et al.^21^ optimized the process parameters of AL7075 using second-order response surface methodology and experimentally verified the consistency between the predicted and experimental values of the response prediction model. Asiltirk et al.^22^ analyzed the effects of feed rate, tool radius of arc, and back engagement on the surface roughness in the turning process as an evaluation metric using Taguchi's methodology and response surface methodology. Rashmi et al.^23,24^ systematically investigated the effect of process parameters on milling force, surface roughness and energy consumption during milling of AA6061 and determined the optimum machining parameters using response surface method and particle swarm optimization algorithm.

Based on the aforementioned studies, the gas cylinder forming process has attained greater maturity, with necking-spinning becoming the dominant production method. During the necking-spinning process, one end of the billet with uniform wall thickness undergoes a certain degree of thickening in the mouth region. However, in the case of gas cylinders with considerable differences in wall thickness between the mouth and the cavity, necking-spinning alone cannot achieve the ideal level of thickening.

For this reason, the research purpose of this paper aims to solve the manufacturing process difficulties of thin-walled and thick-mouthed seamless gas cylinders, and puts forward a boring-reducing neck spinning step-by-step scheme for the overall forming of thin-walled and thick-mouthed seamless gas cylinders, and analyzes the neck-spinning of the cylinders by using finite-element analysis methods, and discusses the stress–strain distributions and geometrical dimensional changes of the cylinders in the process of spinning; and at the same time, through the central composite experimental method involving a number of groups of experiments and carrying out Numerical simulation is carried out to study the effects of spindle speed, friction block working angle and friction coefficient on the forming results, and the second-order response surface method is used to establish the relationship between each process parameter and optimization variables, and then multi-objective optimization is carried out by the NSGA-II algorithm to obtain the optimal solution of Pareto, and the simulation results are finally compared with the experimental results and analyzed.

## Proposal for integral forming of thin-walled and thick-mouthed seamless gas cylinders

### Analysis of forming difficulties

**Figure 1:**
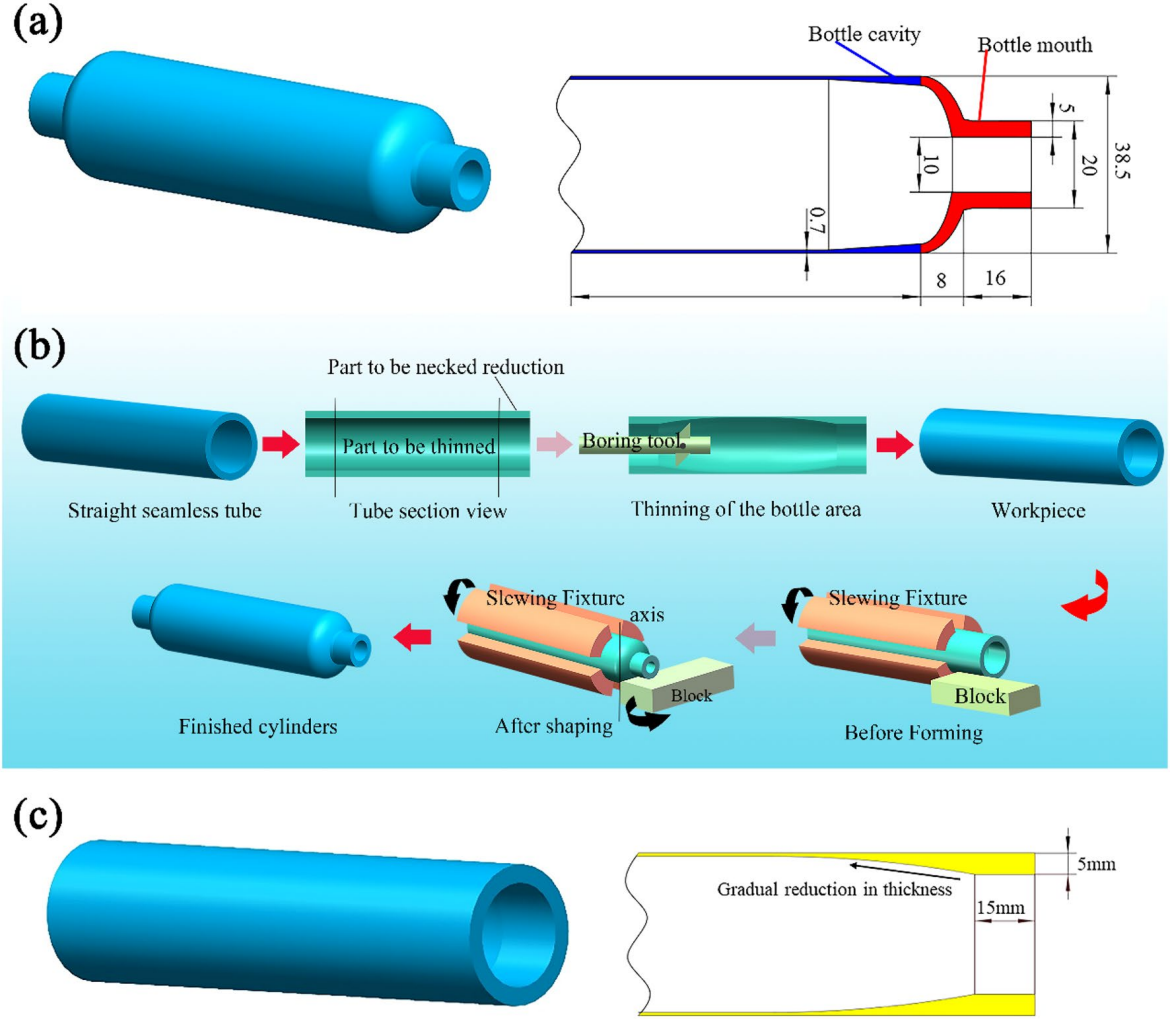
(**a**) Geometry of thin-walled, thick-mouthed seamless gas cylinders. (**b**). Boring-necking spinning step-by-step forming method. (**c**) Geometry of straight seamless tube after boring.

The geometric structure of a thin-walled, thick-mouthed seamless gas cylinder is shown in Fig. 1a. The cylinder was made of 2Cr13 martensitic stainless steel and had a total length of 300 mm, consisting of the cavity and the mouth. The cylinder had a wall thickness of 5 mm at both ends of the mouth and 0.7 mm in the cavity, resulting in a ratio of over 7 between the two wall thicknesses.

The formability of thin-walled, thick-mouthed seamless gas cylinders could be analyzed from both structural and material aspects. Firstly, from a structural formation perspective of gas cylinders, the ratio of wall thickness between the cylinder's mouth and the cavity exceeded seven times, making it challenging to thicken the cylinder's mouth solely through a neck reduction spinning process. Secondly, there was a transition zone with gradually increasing wall thickness between the bottle body and the mouth of the bottle, and the cylinder's cross-section may pose a potential hazard by leading to cracking during the formation process. Finally, 2Cr13 stainless steel exhibits high yield strength and severe hardening during forming, which also results in higher deformation resistance and increases the risk of cracking.

### New method

Considering the large variability of the wall thickness between the cavity and the mouth of a thin-walled, thick-mouthed seamless gas cylinder, solely using a single processing method is not possible.

As such, a step-by-step boring-necking-spin-forming scheme for gas cylinders was proposed in the present study. The flow chart of the new scheme is shown in Fig. 1b. The two-step processing scheme was proposed for achieving desired wall thickness characteristics of thin-walled, thick-mouthed seamless gas cylinders.

- In the first step, according to the overall geometry of the gas cylinder, a straight seamless tube is selected as raw material and divided into two areas: the part of the mouth to be necked reduction and the part of the cavity to be thinned. Boring tools are used to thin the inner wall of the cavity to the target wall thickness, so as to achieve a thin-walled cavity, while retaining the wall thickness in the mouth area to meet the required thickness after forming. Notably, if there is a section with gradually decreasing wall thickness between the mouth and the cavity of the gas cylinder blanks, a workpiece with thicker wall thickness at both ends and thinner wall thickness in the middle should be formed (as shown in Fig. 1c).
- In the second step, both ends of the workpiece are processed separately by means of the necking-spinning process to finally form a thin-walled, thick-mouthed seamless gas cylinder.

**Figure 2:**
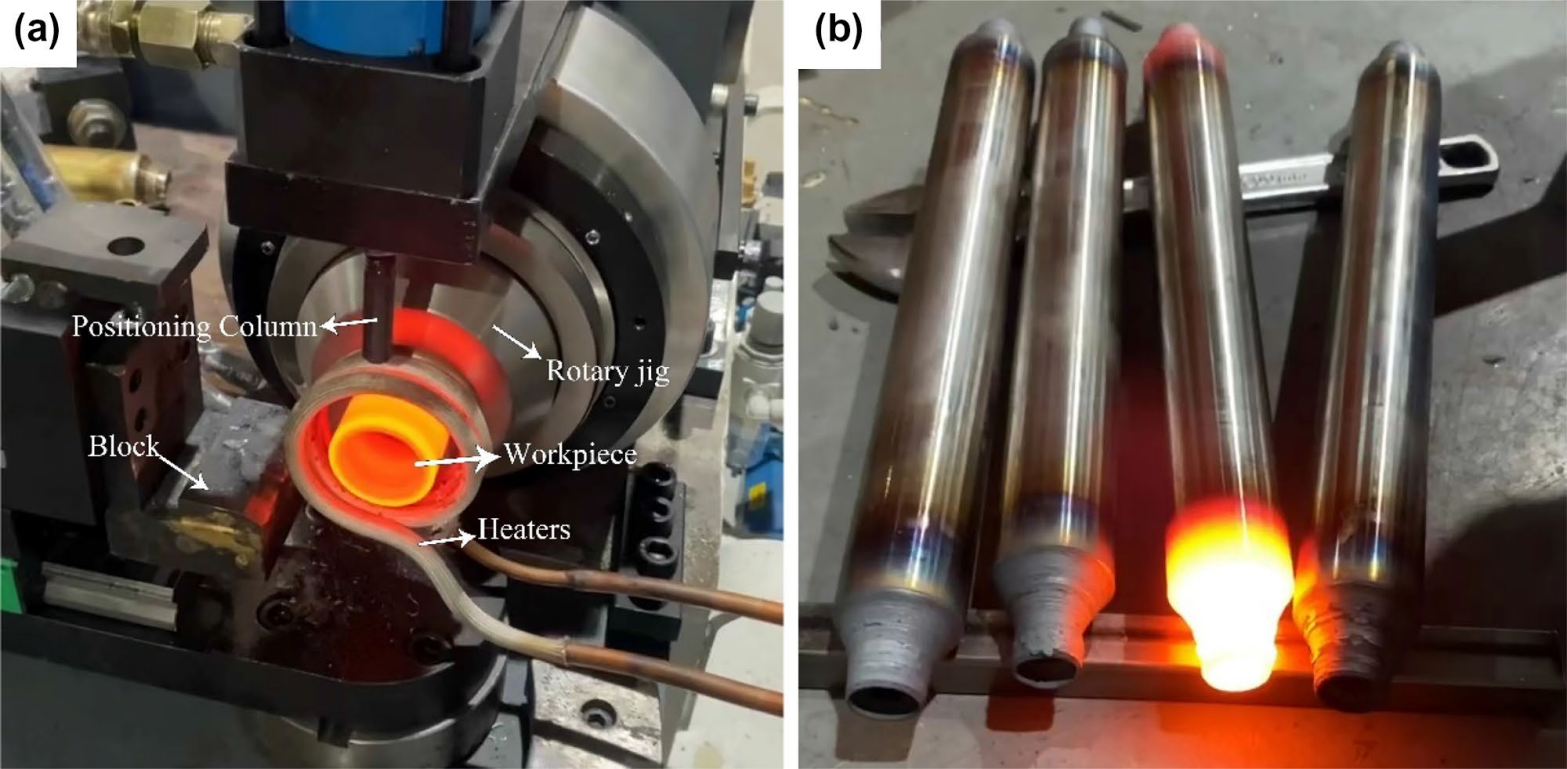
(**a**) Experimental setup; (**b**) finished product processed.

The neck reduction spinning of gas cylinders can be formed by multi-pass spinning with rolls or by spinning with friction blocks of a certain type of surface. The multi-pass spin-forming process for rolls is more complex and has high production requirements. When the friction block is used for spinning, the entire surface of the friction block is in contact with the billet, the contact area is large, and the required forming force is large. Under such conditions, one-time forming can be achieved, and there will be a better thickening effect on the mouth of the gas cylinder, which is suitable for small size workpieces. Owing to the simplicity and efficiency of the process, the friction block spinning method was adopted for processing of the gas cylinder blanks in the present study. Figure 2a shows the experimental setup for gas cylinder forming and a photo of the processed finished product.

### Experimental procedure

The experimental process of neck-spinning of gas cylinders mainly includes the following steps.

- First, the workpiece is clamped in the rotary jig, and the workpiece is exposed to the necked portion using a positioning post at a certain distance.
- Secondly, after the mandrel rotates at high speed, the part of the workpiece to be necked and rotated is heated to the required temperature for forming using a high-frequency induction heater.
- Finally, the position of the friction block is adjusted according to the amount of necking needed to shape the gas cylinder's mouth, and the friction block is not rotated more than 90° around the axis of rotation, resulting in the final formation of the gas cylinder's mouth.

The described steps are repeated for the other end for neck spinning so as to finally form the finished gas cylinder, as shown in Fig. 2b.

### Finite element modeling

**Figure 3:**
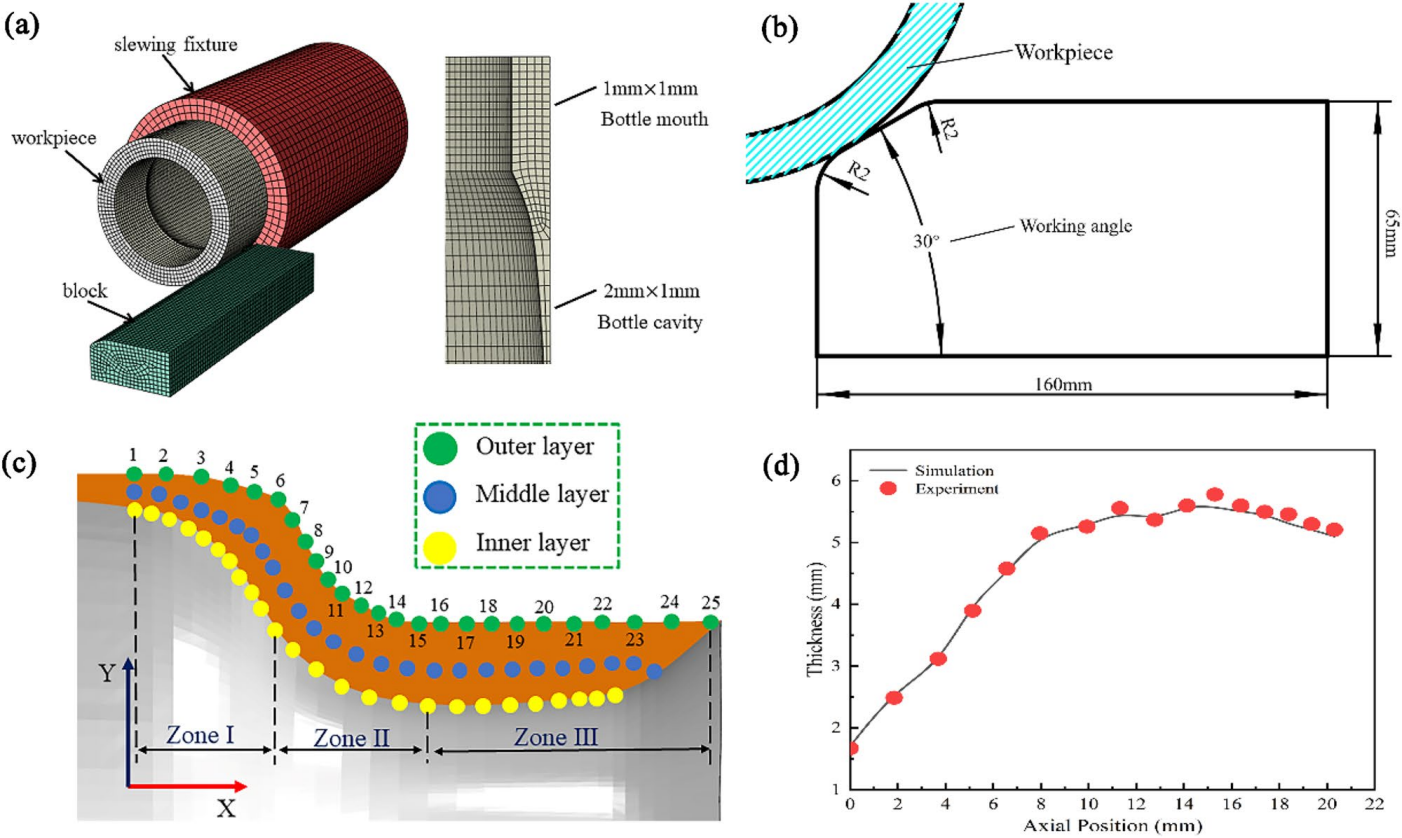
(**a**). Finite element model. (**b**) Friction block working angle diagram. (**c**) Feature node diagram for different thickness layers. (**d**) Comparison of thickness distribution achieved from FE model with experiment.

To investigate the law of neck-spin forming of thin-walled thick nozzle seamless gas cylinders, a 3D finite element model was developed based on ABAQUS software. Additionally, due to the symmetry of the workpiece itself, a 1/2 finite element model was established, as shown in Fig. 3a. In the model, the slewing fixture and friction block were defined as a rigid body, the workpiece was set as a deformable body, and the unit body type was C3d8r.

Initially, the overall mesh size was 2 mm × 1 mm, and to ensure the forming accuracy and simulation efficiency, the mesh of the bottle mouth area was further refined to 1 mm × 1 mm, with the total number of meshes being 29,000. To improve the calculation efficiency, the mass scaling factor was selected as 1000, and the ratio of kinetic energy to internal energy was less than 10% in more than 90% of the spinning process. As such, the simulation was stable.

The material properties are shown in Table 1 and these values were extracted from the study by Ge and Misra et al.^25,26^. These values were obtained through a series of thermal compression tests performed on the material. The main process parameters used in the finite element model are given in Table 2, which were set to be consistent with the actual working conditions.The mandrel speed, friction block working angle, and friction factor were temporarily set to 480r/min, 30° and 0.3, respectively. The schematic diagram of the friction block working angle is shown in Fig. 3b.

**Table 1:** Material parameters^18,19^.

Parameters	Temperature (℃)	Density ρ ($\mathrm{kg}/{\mathrm{m}}^{3}$)	Young’s modulus E (GPa)	Poisson’s ratio $\nu$	Yield strength ${\sigma }_{s}$ (MPa)
Values	950	7500	103	0.33	146

**Table 2:** Parameters of FE models.

Parameters	Values
Necking volume (mm)	10
Mandrel speed (r/min)	480
The working angle of the friction block (°)	30
Friction block speed (r/min)	56.52
Friction factor between friction block and workpiece	0.1

To facilitate the investigation into the deformation of the bottle mouth, the bottle mouth was divided into three deformation zones. Zone I is the portion close to the bottle cavity, Zone III is the free end portion, and Zone II (the current contact area) is the portion between Zone I and III. Moreover, 25 mesh nodes were selected as feature nodes along the axial direction on each of the three thickness layers. The positions of the feature nodes are shown in Fig. 3c. The comparison of the thickness distribution obtained through the finite element model with the experimental results is depicted in Fig. 3d. Obviously, there is a good agree-ment between the simulation results and the experimen-tal ones.

## Analysis of the forming process

### The stress and strain distribution during the forming process

**Figure 4:**
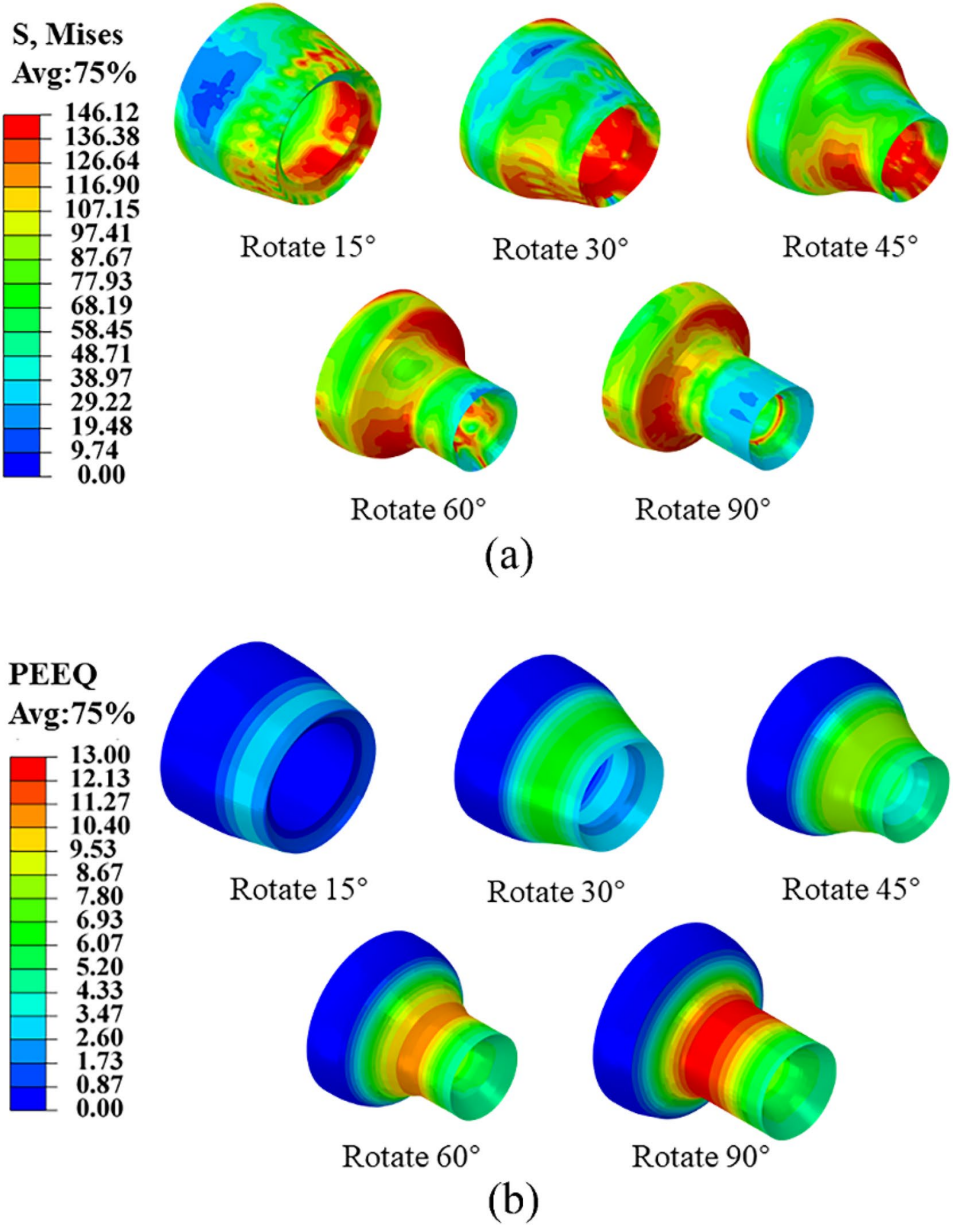
(**a**) Stress distribution at different rotation angles. (**b**) Strain distribution at different rotation angles.

Figure 4 shows the stress and strain distribution of the gas cylinder mouth at different stages of the spinning process. The neck spinning process of a gas cylinder can be divided into three stages: Stage 1, the neck of the bottle mouth forming; Stage 2, the cylinder mouth forming, also the neck shape trimming process; and Stage 3, the cylinder bending forming, but also the neck and mouth finishing process.

Figure 4a shows that the equivalent stress is predominantly distributed within the vicinity of the contact area, with the maximum stress concentration observed at the bottleneck region and the friction block's contact position. Moreover, as the distance from the contact region increases, the equivalent force diminishes. Such local plastic deformation features of the friction block's neck rotation spinning govern the observed phenomenon. In the spinning process, the contact area increases first, and then gradually decreases after reaching a certain peak, so the distribution range and size of the equivalent force also show a trend of increasing and then decreasing. When the free end of the bottle mouth is no longer in contact with the friction block, the free end of the bottle mouth is less constrained and its stress value becomes small.

Figure 4b shows that the equivalent effect variation was distributed hierarchically along the axial direction, and the size of the equivalent effect variation was equal in the same circumferential direction. With the gradual feeding of the friction block, the equivalent variation of the bottle mouth increased until the end of spinning. The maximum equivalent effect variation value was located at the neck of the bottle curvature, which indicates that there was a large metal flow, with the potential occurrence of cracks and buildup (as shown in Fig. S1).

### Geometric changes (thickness, elongation, and profile)

Xia et al.^27^ explored tube neck spinning and reported that the thickness deviation between the actual dimensions of the workpiece and the nominal dimensions of the part must be satisfied. After the neck-spinning of the cylinder mouth, it is essential to create threaded holes in its inner wall. However, due to the limited thickness of the mouth, the tolerance of the fit may be impacted. Therefore, it is crucial to ensure that the thickness of the mouth meets the required wall thickness.

**Figure 5:**
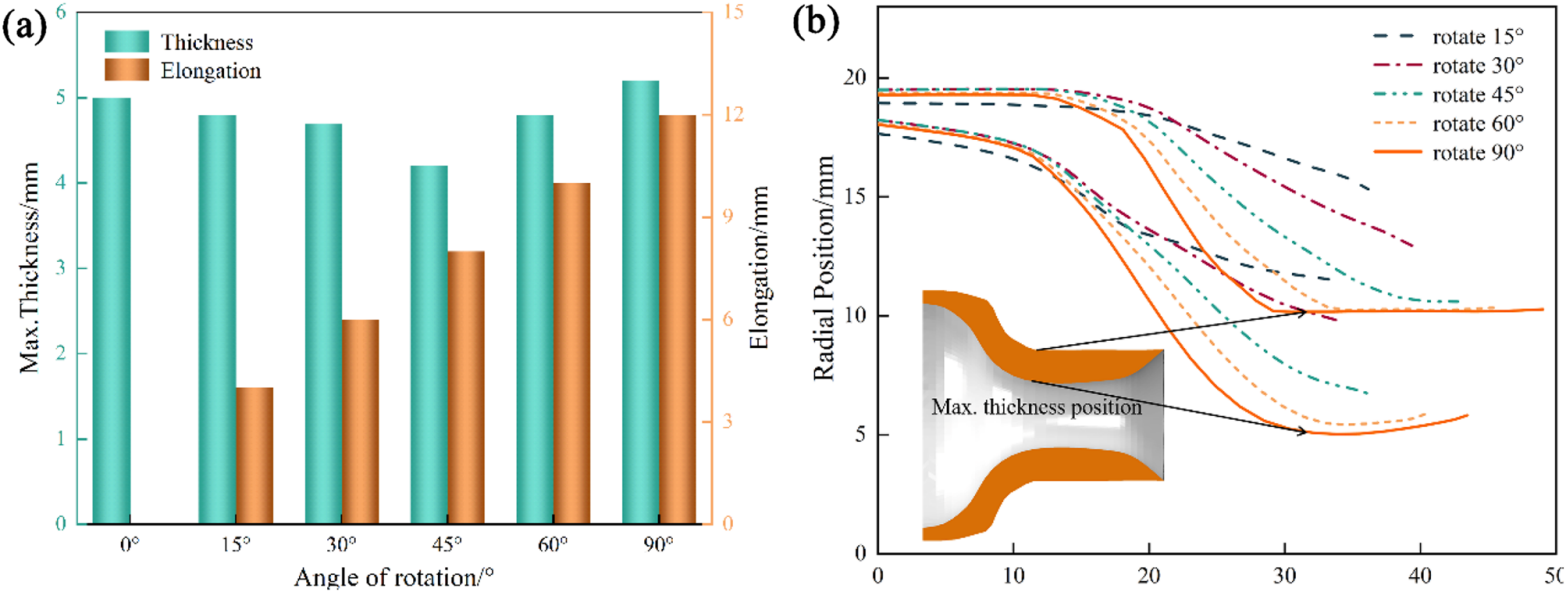
(**a**) Thickness and elongation of bottle mouth at different forming stages. (**b**) The inner and outer profile of the bottle mouth at different forming stages.

Figure 5a shows the thickness and elongation of the bottleneck at different stages of the spinning process. An observation can be made the elongation of the bottleneck increased from the beginning to the end of the spinning, while the wall thickness exhibited a process of decreasing and then increasing, and the final wall thickness increased compared with the original wall thickness.

Figure 5b shows the internal and external profiles of the bottle mouth during the rotary feeding of the friction block. An observation can be made that as the spinning proceeded, the mouth of the bottle was gradually compressed to form a bend and the elongation band became more pronounced. The maximum wall thickness value of the bottle mouth was 5.2 mm, which occurred at the inflection point, as shown in the lead in Fig. 5b. The minimum thickness value appeared at the edge of the free end of the bottle mouth area, and there was an obvious thinning band at the free end of the bottle mouth. The wall thickness reduction at the edge of the free end became more serious as the friction block was gradually rotated and fed.

### The plastic strain distribution of different thickness layers

**Figure 6:**
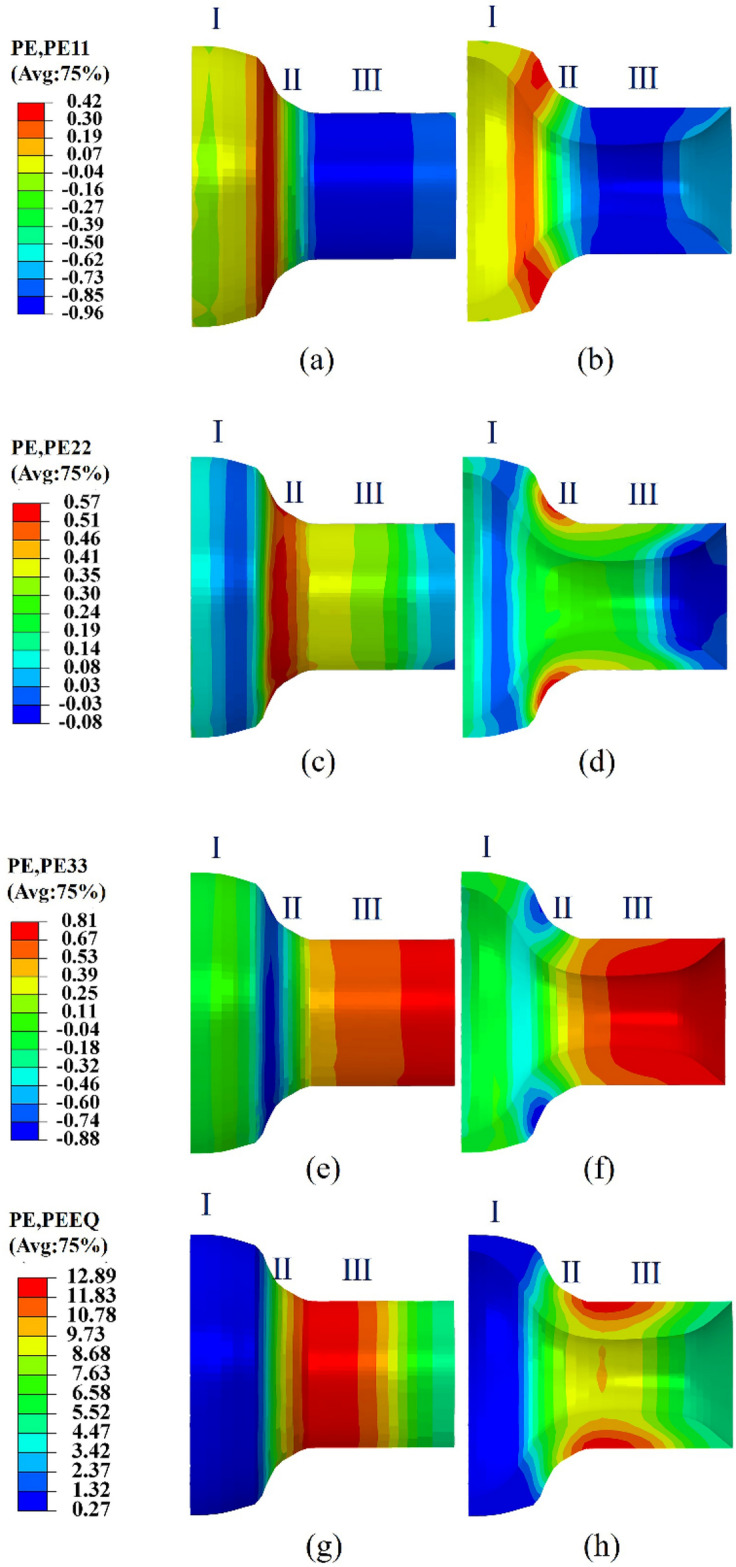
Strain distribution during bottle mouth forming. (**a**) Radial strain of the surface, (**b**) radial strain of the axial cross-section, (**c**) circumferential strain of the surface, (**d**) circumferential strain of the axial cross-section, (**e**) axial strain of the surface, (**f**) axial strain of the axial cross-section, (**g**) equivalent strain of the surface, (**h**) equivalent strain of the axial cross-section.

**Figure 7:**
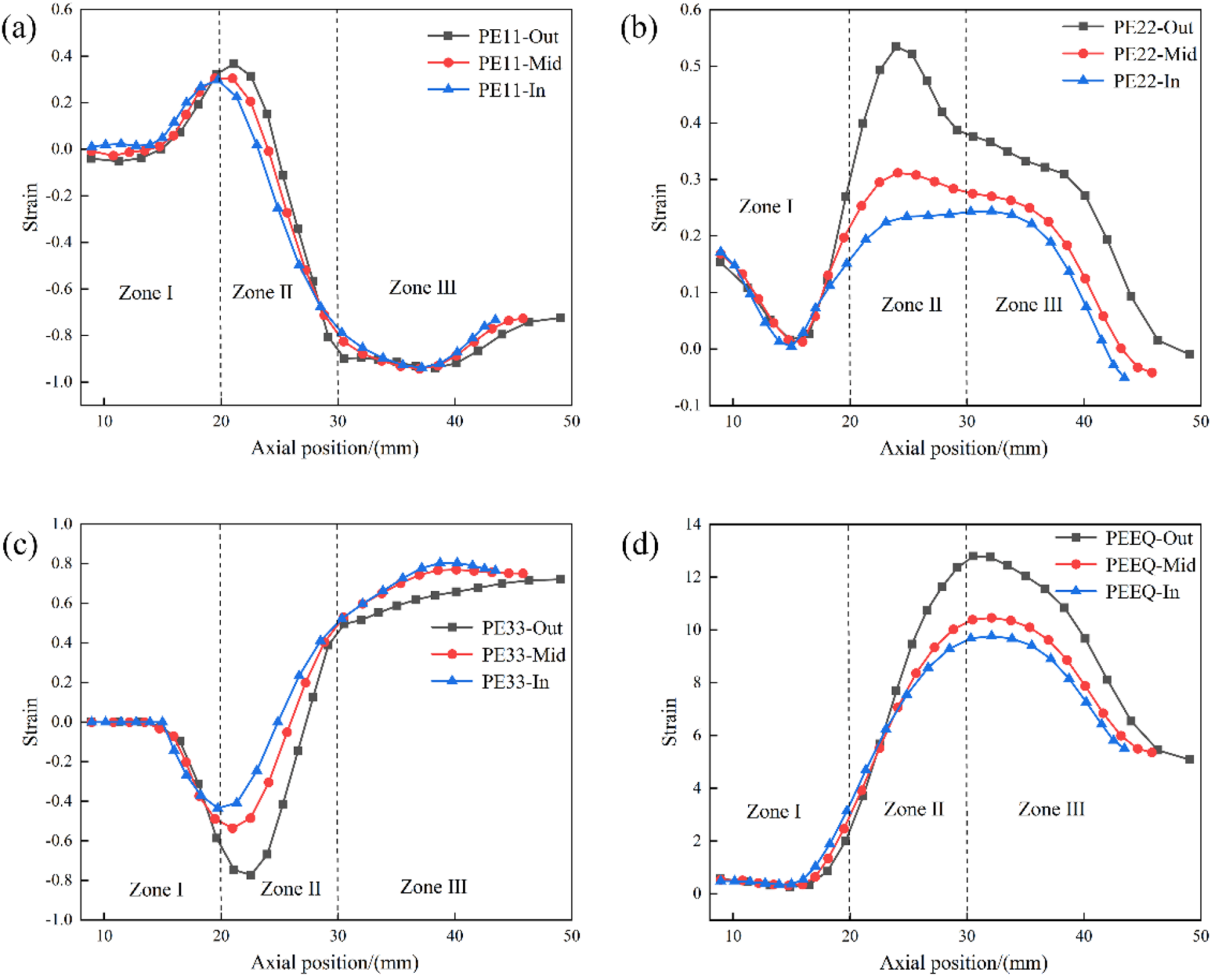
Strain distribution along the axial direction for the three thickness layers. (**a**) Radial strain, (**b**) circumferential strain, (**c**) axial strain, (**d**) equivalent strain.

With the rotary feed of the friction block, under the high speed operation of the spindle, with the help of air mold forming, the trajectory of the friction block movement so that it is always parallel to the surface of the workpiece, and keep the flat section and the workpiece is in complete contact with the mouth of the bottle gradually compressed to form a bending, resulting in a large plastic deformation, so it is necessary to analyze the different regions of the bottle mouth, Fig. 3c shows the different regions of the bottle neck. To analyze the variation of the bottle mouth thickness and elongation, the radial, circumferential, axial, and equivalent plastic strains at the gas bottle mouth were examined, as shown in Fig. 6. In manufacturing certain pressure vessels using the tube spinning process, Zoghi and Fallahi Arezoodar^9^, highlighted the existence of thickening, thinning, elongation, compression, bending, and shearing in the deformation zone during the neck spinning process of the bottle mouth. To further investigate the strain state of the bottle mouth, the plastic strain distribution on each thickness layer in Fig. 3c was examined. Figure 7a–d depict the distribution of radial, circumferential, axial, and equivalent variation for different thickness layers, respectively.

From Fig. 6, an observation can be made that the strain distribution of each thickness layer within Zones I and III was relatively uniform, and the differences between thickness layers were mainly reflected in Zone II (contact area).

Figure 6a, b depict the distribution of the radial strain, which is usually negative due to the necking behavior of the free end of the workpiece. An observation can be made that in Zone III, the strain state was reflected as a compression strain. Figure 6b shows that positive strain existed in Zone II and the radial positive strain decreased from the outer layer to the inner layer. Combined with Fig. 7a, a conclusion could be drawn that there was no significant difference in radial strain between the three thickness layers in the bottle mouth region, indicating that the decrease in diameter during necking rotation was uniform between the thickness layers. Biglari^28^ reported a similar pattern of radial strain when rotating the plate with a hemispherical mandrel.

Figure 6c, d show the distribution of circumferential strains, which were consistently reflected as positive strains (tensile strains) in different zones between the three thickness layers. As seen in Fig. 6d, the positive strain in Zone II decreased gradually along the outer layer to the inner layer. While directly subjected to the force of the friction block, the outer layer of the tube was still undergoing positive circumferential strain (contact area) as a result of the constant volume during plastic deformation. In combination with Fig. 7b, the strain values of the three thickness layers in Zone II gradually increased, implying an increase in thickness. The strain value of the thickness layer in Zone III gradually decreased, and the circumferential strain at the edge of Zone III sharply decreased, which indicates a slight thinning in the thickness direction.

Figure 6e, f show the distribution of the axial strain. The axial strains in the three thickness layers in Zones I and II were reflected as negative strains (compressive strains), and the axial strains in Zone III were positive (tensile strains). In combination with Fig. 7c, a conclusion could be drawn that the negative strain in Zone I gradually increased, which was caused by the local force of the friction block causing the bending of each thickness layer by axial compression. The negative strain in Zone II gradually decreases and then rapidly changes direction. The axial strain in each thickness layer in Zone III was in a tensile state after the direction of strain transition, and the strain value gradually increased towards the free end. The reduction of circumferential strain in Zone III was a result of volume constancy, which compensated to some extent the positive axial strain of volume constancy. The bending due to compressive strain along the axial direction in Zone II and the axial extension in Zone III together amplified the positive strain. Such findings are consistent with the deformation pattern of Zoghi and Fallahi Arezoodar^9^ in their research on neck spinning. The excessive elongation of the outer metal at the rim in Zone III was caused by the unconstrained material at the rim of the bottle mouth, which is consistent with the behavior mentioned by Hamed et al.^19^ in their research on tube spinning.

**Figure 8:**
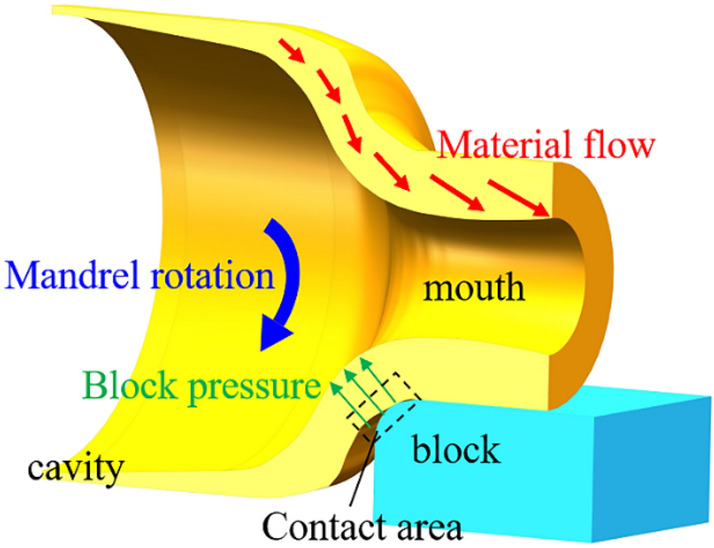
Schematic diagram of material flow in the spinning process.

According to the principle of volume invariance, due to the necking behavior of the bottle mouth, the metal flowed more along the axial direction at the free end, then the flow in the circumferential direction had to be reduced. Moreover, the radial flow of the material gradually slowed down along the wall thickness direction, as shown in Fig. 8. Therefore, the deformation of the gas cylinder mouth was mainly reflected in the axial elongation, while the thickening in the thickness direction was small.

Figure 6g, h show the distribution of the equivalent strain. As expected, there was a larger equivalent strain in Zone II. From Fig. 6h, an observation can be made that the values of the equivalent strain in the same circumferential direction remained consistent, and the equivalent strain in Zone II gradually decreased from the outer to the inner layers. As can be seen in Fig. 7d, in the axial direction, the value of the equivalent strain increased and then decreased. The outer layer strain had a larger growth rate. The maximum equivalent strain value was located at the intersection of Zone III and Zone II (near node 14). Such findings indicate that there was a large amount of metal flow here and the strain gradient was more intense, which could be attributed to the presence of curvature mutations in Zone II.

## Response surface and parametric equations for spinning process parameter optimization

The central composite design is a commonly used method for response surface analysis, which is based on the two-level full-factor partial test design. It allows a finite set of sample points to be obtained in the design domain, reducing the number of tests^29^. The central composite experimental design response surface methodology (RSM) is a combination of mathematical and statistical techniques used to develop, improve and optimize processes. Using the central composite experimental method, multiple sets of experiments were designed and numerically simulated to analyze the effects of each process parameter on the spinning results. Response surface methodology (RSM) is an optimization method that integrates experimental design and mathematical modeling, which effectively reduces the number of experiments and allows for the examination of interactions between influencing factors, and the mapping relationship between each process parameter and the optimization variables was established by means of the response surface method^30^. The resulting mapping relations were used to perform multi-objective optimization using the NSGA-II algorithm and obtain the Pareto optimal solution, so as to optimize the process parameters and improve the quality of gas cylinders^31^.

### Central composite experimental design

The maximum stress, and minimum thickness were selected as the optimization objectives, and the three parameters of spindle speed, friction block working angle and friction coefficient were taken as the influencing factors of the central composite experimental design to construct the response surface model. Such means could avoid the stress concentration during the forming process of the gas cylinder mouth causing damage to the workpiece and further control the wall thickness of the mouth. Table 3 shows the range of values for each parameter. Table S1 shows the central composite experimental parameters obtained by numerical simulation.

**Table 3:** Range of experimental parameter variation.

Parameters	Max. value	Min. value	Average value
Mandrel speed ${x}_{1}$(r·${\mathrm{min}}^{-1}$)	600	400	500
friction block working angle ${x}_{2}$ (°)	35	25	30
Friction factor ${x}_{3}$	0.1	0.3	0.2

The effects of each variable on the maximum stress and minimum wall thickness and their interactions were calculated using Design-Expert13 software. The relationship between the factors (independent variables) and each response (dependent variable) was modeled by fitting a second-order polynomial equation given by the following equation.

The effects of each variable on the maximum stress and minimum wall thickness and their interactions were calculated using Design-Expert13 software. The relationship between the factors (independent variables) and each response (dependent variable) was modeled by fitting a second-order polynomial equation given by the following equation:

$$Y={\alpha }_{0}+{\alpha }_{1}{X}_{1}+{\alpha }_{2}{X}_{2}+{\alpha }_{3}{X}_{3}+{\alpha }_{12}{X}_{1}{X}_{2}+{\alpha }_{13}{X}_{1}{X}_{3}+{\alpha }_{23}{X}_{2}{X}_{3}+{\alpha }_{11}{{X}_{1}}^{2}+{\alpha }_{22}{{X}_{2}}^{2}+{\alpha }_{33}{{X}_{3}}^{2}$$

in which ${X}_{1}$, ${X}_{2}$, and ${X}_{3}$ are the independent variables in the response surface model parameters, ${\alpha }_{0}$, ${\alpha }_{1}$, ${\alpha }_{2}$, ${\alpha }_{3}$, ${\alpha }_{11}$, ${\alpha }_{12}$, ${\alpha }_{13}$, ${\alpha }_{22}$, ${\alpha }_{23}$, and ${\alpha }_{33}$ are the regression coefficients, and $Y$ is the response function.

### Response surface results

**Figure 9:**
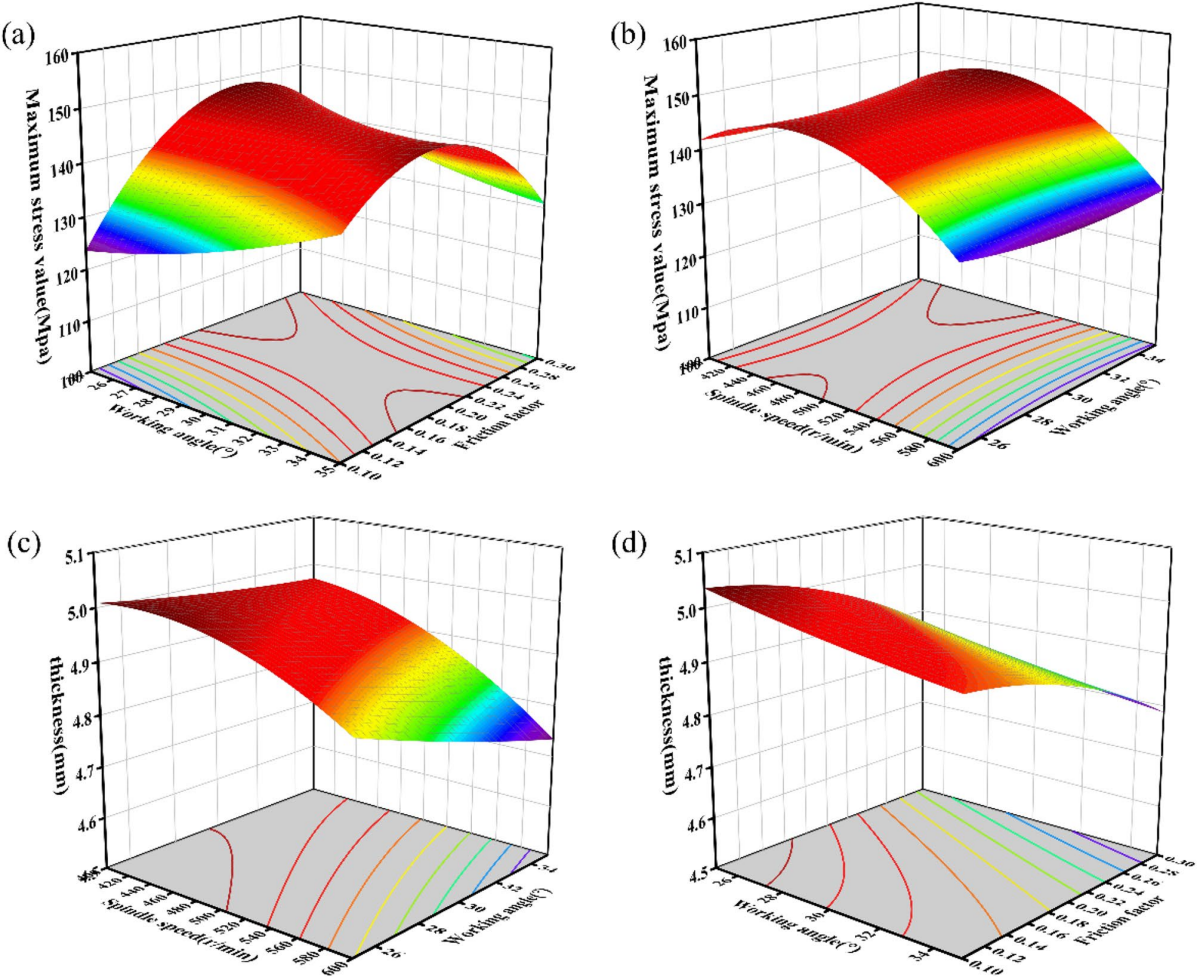
(**a**) Effect of mandrel speed and working angle on maximum stress. (**b**) Effect of working angle and friction factor on maximum stress. (**c**) Effect of spindle speed and working angle on wall thickness values. (**d**) Effect of working angle and friction factor on wall thickness values.

(1) Mapping relationship between a maximum stress and independent variables and response surface model.
  The prediction model of the regression equation for the independent variables ${X}_{1}$, ${X}_{2}$, ${X}_{3}$ and the dependent variable ${Y}_{1}$ (maximum stress) is shown in Eq. (1). Figure 9a shows the response surface of the maximum stress (${Y}_{1}$) with the variation of mandrel speed (${x}_{1}$) and friction block working angle (${x}_{2}$), and Fig. 9b shows the response surface of the maximum stress (${Y}_{1}$) with the variation of friction block working angle (${x}_{2}$) and friction factor (${x}_{3}$).

  1 $${Y}_{1}=-190.43150+0.990726{x}_{1}-0.3416{x}_{2}+1034.84125{x}_{3}-0.001684{x}_{1}{x}_{2}-0.151075{x}_{1}{x}_{3}-14.03200{x}_{2}{x}_{3}-0.000969{{x}_{1}}^{2}+0.067795{{x}_{2}}^{2}-1267.28750{{x}_{3}}^{2}$$
  Figure 9a reflects that the maximum stress decreased with the gradual increase of the spindle speed, which could be ascribed to the increased mobility of the material in the circumferential and axial directions with the increase in spindle speed, rendering a decrease in the internal stress. Figure 9b reflects that the maximum stress increased with the gradual increase in the friction factor, which was due to the increase in the resistance to the flow of the metal at the mouth of the bottle with the increase in the friction factor, and the formation of larger stress. The effect of the working angle on the maximum stress was smaller.
Mapping relationship between minimum thickness and independent variables and response surface model
  The prediction model of the regression equation for the independent variables ${x}_{1}$, ${x}_{2}$, ${x}_{3}$, and the dependent variable ${Y}_{2}$ is shown in Eq. (2). Figure 9c shows the response surface of the wall thickness value (${Y}_{2}$) with the variation of spindle speed (${x}_{1}$) and working angle (X2), and Fig. 9d shows the response surface of the wall thickness value (${Y}_{2}$) with the variation of working angle (${x}_{2}$) and friction factor (${x}_{3}$).
  2 $${Y}_{2}=4.13938+0.004942{x}_{1}-0.003977{x}_{2}-0.245801{x}_{3}-0.000051{x}_{1}{x}_{2}+0.001461{x}_{1}{x}_{3}+0.030487{x}_{2}{x}_{3}-4.62650E-06{{x}_{1}}^{2}+0.00024{{x}_{2}}^{2}-3.19512{{x}_{3}}^{2}$$

Figure 9c, d reflect that the wall thickness value decreased with the increase in spindle speed and friction factor, which could be attributed to the increase in spindle speed and friction factor, as well as the metal flow at the mouth of the bottle being enhanced and retaining less metal the thickness direction. of the increase in working angle tended to lead to a decrease in the wall thickness value, which was because the increase in working angle led to the corresponding increase in contact area between the friction block and the mouth of the bottle, thereby accelerating the material flow along the axial direction leading to a decrease in wall thickness.

**Figure 10:**
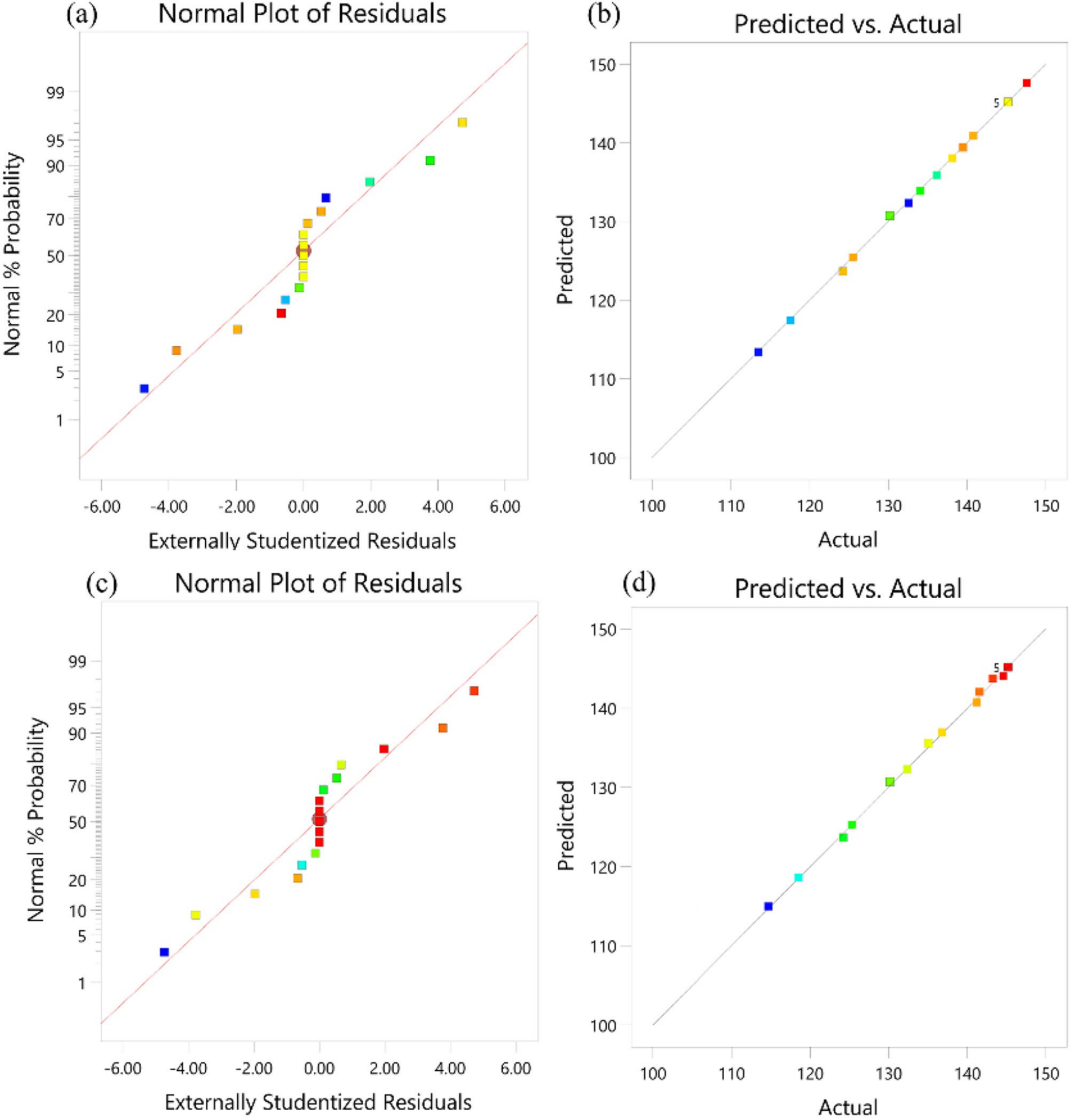
(**a**) Normal plot of residuals for Max. Stress, (**b**) predicted vs. actual for Max. Stress, (**c**) normal plot of residuals for Min. thickness, (**d**) predicted vs. actual for Min. thickness.

The normal plots of residuals for Max. Stress and Min. thickness as demonstrated in Fig. 10a, c are approximately linear, indicating that the residuals are normally distributed and the fittings of regression equations for Max. Stress and Min. thickness are reasonable. The points cluster near by the diagonal line are observed between the predicted and actual values of Max. Stress and Min. thickness in Fig. 10b, d. It suggests that the established model is strongly appropriate for quantitatively describing the influence of various factors on the response of Max. Stress and Min. thickness.

### Multi-objective optimization

According to the parameter equations discussed in "Response surface results" for the multi-objective optimization, the problem to be investigated could be described in the context of the actual situation, where 400 ≤ ${x}_{1}$≤600, 25 ≤ ${x}_{2}$≤35, and 0.1 ≤ ${x}_{3}$≤0.3.

In order to prevent cylinder failure caused by excessive stress at the bottle mouth and to ensure adequate thickness in the same Zone, it was necessary to simultaneously achieve the minimum value of the stress variable ${Y}_{1}$ and the maximum value of the thickness variable ${Y}_{2}$, while keeping the independent variables ${x}_{1}$, ${x}_{2}$, and ${x}_{3}$ within the allowed variation range.

Using Design Gateway for multi-objective optimization, the regression equations were entered into the computer component, the initial values of the design variables ${x}_{1}$, ${x}_{2}$, and ${x}_{3}$ were entered, the upper and lower limits of the variables were set, and the NSGA-II algorithm was selected to solve for all the Pareto solutions. In the Design Gateway window, the scatter plot between the maximum stress ${Y}_{1}$, the thickness increment ${Y}_{2}$ and the independent variables can be observed as shown in Fig.S2a. In Fig.S2a, the Pareto solutions (i.e., Pareto fronts) are uniformly distributed and located at the right fronts of all test points. Fig.S2b shows the distribution of the Pareto optimization solution set on the 3D coordinate axes. By analyzing the distribution of the Pareto optimized solution set on the 3D coordinate axes, an observation can be made that the optimized solution set was mainly concentrated in a specific range, being a significant reference value for the optimization and design of the target quantities. The optimal set of solutions provided by Design in the optimized solution set was the spindle speed of 490.4, a working angle of 34.439°, and a friction coefficient of 0.248.

## Verification of finite element simulation

Based on the experimental setup shown in Fig. 2a, spinning experiments were performed on the workpiece. The process scheme was designed based on the process parameters determined by multi-objective optimization, discussed in "Multi-objective optimization". Finite element simulations and experiments were performed on the process scheme, and the simulation results were compared with the experimental results, as shown in Fig. S3a, b.

Due to the high temperature of the spinning process, there were difficulties in performing real-time measurements, and it was not possible to investigate the deformation by microstructure due to the reversion and recrystallization at high temperatures. As such, a decision was made to use the internal and external contours of the gas cylinder after molding, as well as the wall thickness distribution to verify the simulation. Several researchers^32,33^ used a similar approach to perform a validation of the reliability of the model.

A comparison of the wall thickness distribution and the cross-sectional profile dimensions is shown in Fig. S3c, d, respectively. The maximum difference between the measured thickness from the workpiece and the predicted thickness obtained from the simulation was about 8.02%, being within the allowable range. In the experiments and simulations, the minimum wall thicknesses were 5.2 and 5.014 mm, respectively. In the actual working conditions, with heat dissipation and local heating of the supplemental heat device, the temperature in the deformation area of the billet was not uniform, while the simulated temperature was constant in the simulation, thereby resulting in errors between the simulation and the actual machining.

Such results indicate a good agreement between the simulation and the experiment. In conclusion, the developed finite element model is reliable and the cross-sectional profile of the part is essentially consistent with the target shape after spinning. The deviations of both wall thickness and profile are within acceptable limits. Therefore, the proposed scheme can be considered an effective method to achieve thin-walled and thick-mouthed gas cylinder forming.

## Conclusions

In the present study, a step-by-step boring-neck spinning process scheme was developed for forming thin-walled and thick-mouthed seamless gas cylinders. Based on finite element analysis and experimental studies, the following conclusions were drawn:

1. In the process of neck-spinning of gas cylinders, there was a large stress in the mouth region and the axial elongation of the mouth was more significant, while there was a smaller thickening effect in the thickness direction.
2. The excessive elongation of the metal in the outer layer at the edge of the free end of the bottle neck (zone III) during neck-down spinning is caused by the unconfined material at the edge of the bottle neck, which is consistent with the behavior mentioned by Hamed et al. researchers in their pipe spinning studies. In the case of constant volume, the necking behavior of the bottle neck makes the metal flow more in the axial direction and then the flow in the circumferential direction has to be reduced.
3. As the mandrel speed, friction block working angle, and friction factor increased, the wall thickness of the bottle mouth decreased, while the maximum stress increased rapidly. Considering the influence of process parameters on the aforementioned factors, a process parameter scheme was determined as follows: spindle speed of 490 r/min, friction block working angle of 34°, and friction coefficient of 0.25.
4. Experimental studies revealed that the desired thin-walled, thick-mouthed seamless gas cylinders can be formed using a step-by-step boring-necking spinning scheme and defined process parameters. The minimum wall thickness of the gas cylinder mouth in the experiment was 5.2 mm, and the geometric profile accuracy as well as the wall thickness after forming was within the error range. As such, the proposed step-by-step boring-neck-spinning scheme may become an advanced processing technology for forming thin-walled and thick-mouthed gas cylinder liners. The accurate prediction of the inner and outer contour molding of the bottle mouth area can be studied in depth in the future to further overcome the molding barriers of thin-walled and thick-mouthed gas cylinder liners.

### Supplementary Information

Supplementary Information.

## Data Availability

During the current study, the datasets in Table1 Material parameters were available from the corresponding author upon reasonable request. The rest of the data generated or analyzed are included in this publication and in the supplementary information.
