# Supplementary material for: Research on the integral forming process of thin walled and thick mouth seamless gas cylinders
Source: Sci Rep. 2023 Oct 9;13:17021. doi: 10.1038/s41598-023-44377-z (PMC10562450; doi:10.1038/s41598-023-44377-z)
Supplement: Supplementary file 1 — Supplementary Information. [file 41598_2023_44377_MOESM1_ESM.docx]

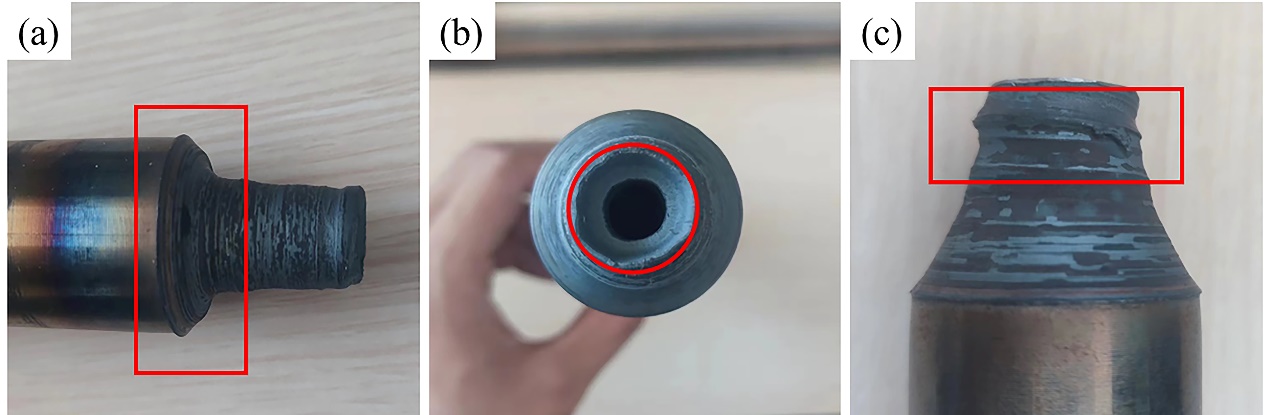


Fig.S1. Forming defect at the bottle mouth(a) Stacking (b) Circularity difference


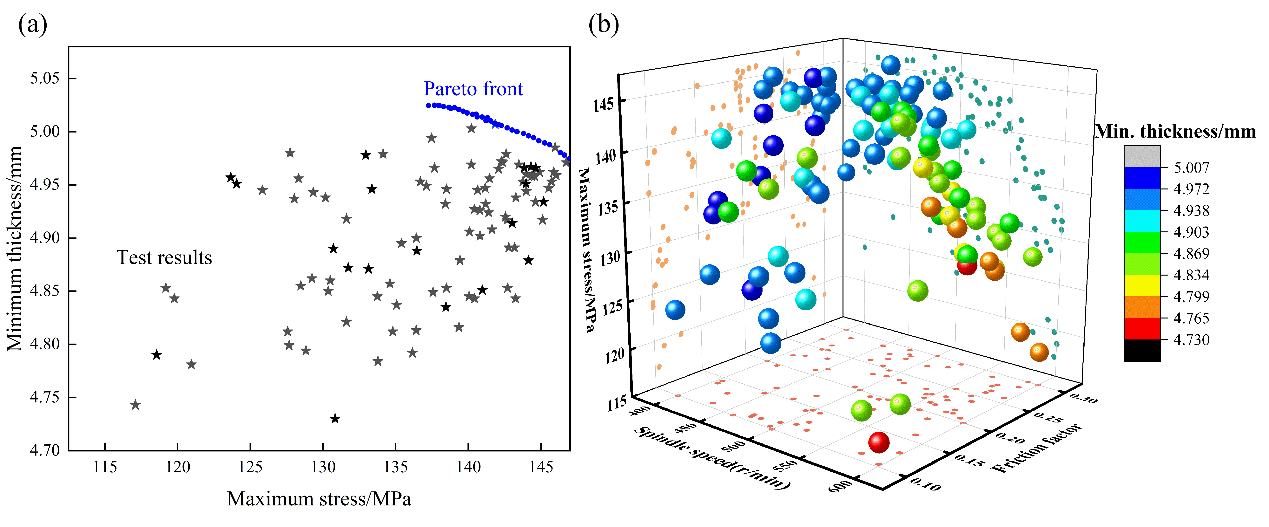


Fig.S2. (a) Distribution of optimal solutions for maximum stress and minimum wall thickness

(b) Distribution of optimal solutions for stress, wall thickness, spindle speed, and friction coefficient on 3D view


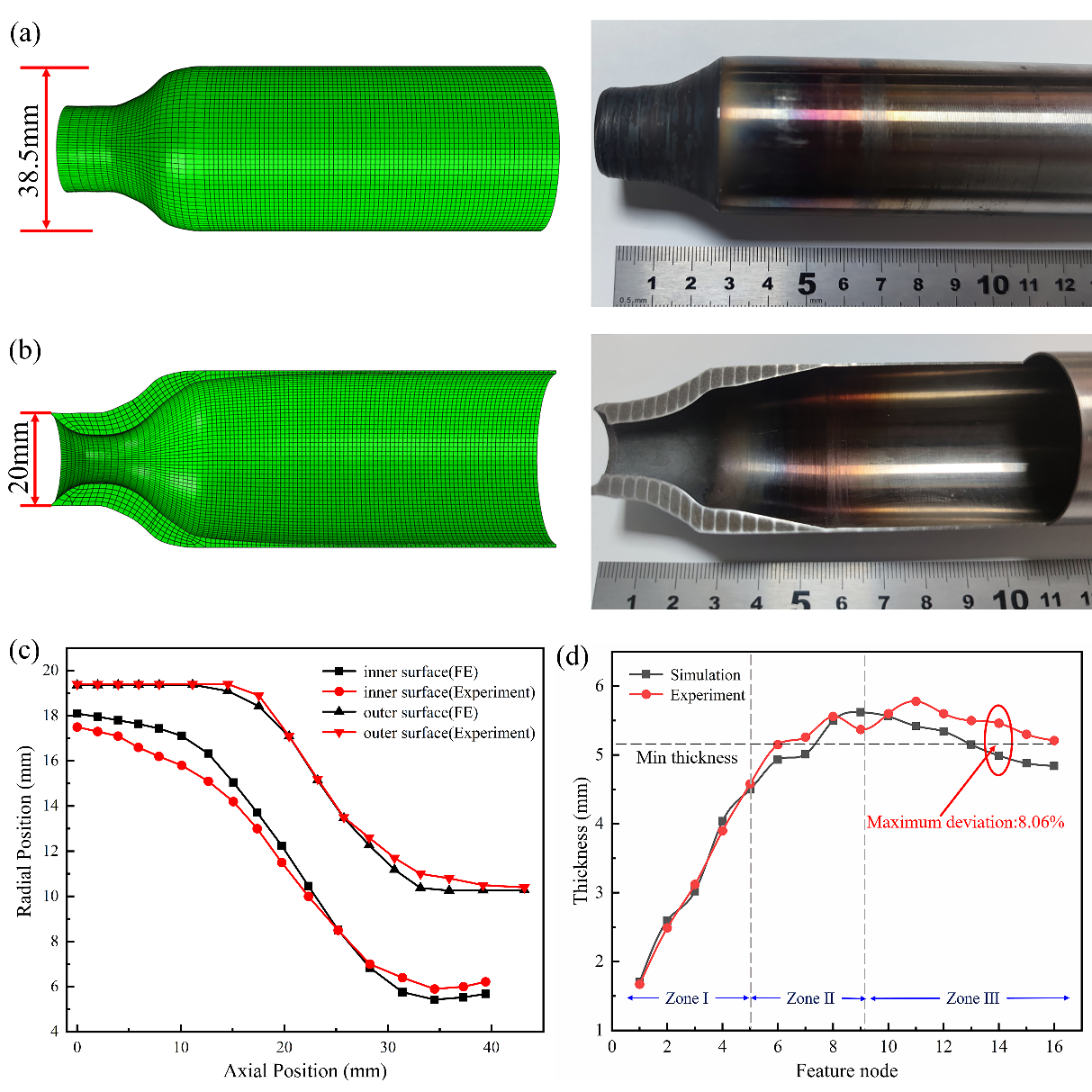


Fig. S3. (a) Experimental and simulation results (b) Sectional view

(c) Comparison of external dimensions (d) Comparison of wall thickness distribution

Table S1 Central composite experiment parameters

| number | Mandrel speed $x_{1}$(r·$\min^{-1}$) | friction block working angle $x_{2}$(°) | Friction factor $x_{3}$ | Max. stress $Y_{1}$(MPa) | Min. thickness $Y_{2}$(mm) |
| --- | --- | --- | --- | --- | --- |
| **1** | 400 | 25 | 0.2 | 166.5 | 5.003413 |
| **2** | 600 | 25 | 0.2 | 178.1 | 4.873272 |
| **3** | 400 | 35 | 0.2 | 163.3 | 4.965969 |
| **4** | 600 | 35 | 0.2 | 181.5 | 4.733387 |
| **5** | 400 | 30 | 0.1 | 189.1 | 4.958697 |
| **6** | 600 | 30 | 0.1 | 177.4 | 4.739414 |
| **7** | 400 | 30 | 0.3 | 154.6 | 4.943488 |
| **8** | 600 | 30 | 0.3 | 164.6 | 4.78265 |
| **9** | 500 | 25 | 0.1 | 212.2 | 4.952946 |
| **10** | 500 | 35 | 0.1 | 202.0 | 4.829198 |
| **11** | 500 | 25 | 0.3 | 160.0 | 4.956963 |
| **12** | 500 | 35 | 0.3 | 161.8 | 4.894189 |
| **13** | 500 | 30 | 0.2 | 180.0 | 4.934278 |
